# Links between melanoma germline risk loci, driver genes and comorbidities: insight from a tissue‐specific multi‐omic analysis

**DOI:** 10.1002/1878-0261.13599

**Published:** 2024-02-03

**Authors:** Michael Pudjihartono, Evgeniia Golovina, Tayaza Fadason, Justin M. O'Sullivan, William Schierding

**Affiliations:** ^1^ Liggins Institute The University of Auckland New Zealand; ^2^ The Maurice Wilkins Centre The University of Auckland New Zealand; ^3^ Australian Parkinson's Mission Garvan Institute of Medical Research Sydney Australia; ^4^ MRC Lifecourse Epidemiology Unit University of Southampton UK; ^5^ Singapore Institute for Clinical Sciences Agency for Science, Technology and Research (A*STAR) Singapore City Singapore

**Keywords:** comorbidity, epigenetics, gene regulation, genetics, melanoma

## Abstract

Genome‐wide association studies (GWAS) have associated 76 loci with the risk of developing melanoma. However, understanding the molecular basis of such associations has remained a challenge because most of these loci are in non‐coding regions of the genome. Here, we integrated data on epigenomic markers, three‐dimensional (3D) genome organization, and expression quantitative trait loci (eQTL) from melanoma‐relevant tissues and cell types to gain novel insights into the mechanisms underlying melanoma risk. This integrative approach revealed a total of 151 target genes, both near and far away from the risk loci in linear sequence, with known and novel roles in the etiology of melanoma. Using protein–protein interaction networks, we identified proteins that interact—directly or indirectly—with the products of the target genes. The interacting proteins were enriched for known melanoma driver genes. Further integration of these target genes into tissue‐specific gene regulatory networks revealed patterns of gene regulation that connect melanoma to its comorbidities. Our study provides novel insights into the biological implications of genetic variants associated with melanoma risk.

## Graphical abstract

This study integrates melanoma‐associated GWAS (genome‐wide association study) SNPs with tissue‐specific epigenomic, Hi‐C (3D genome organization), and eQTL (expression quantitative trait loci) data. We identified genes targeted by these SNPs and their protein interactions (enriched in known melanoma drivers). By integrating these genes into gene regulatory networks, we link melanoma risk to its comorbidities, offering new insights into its genetic basis.

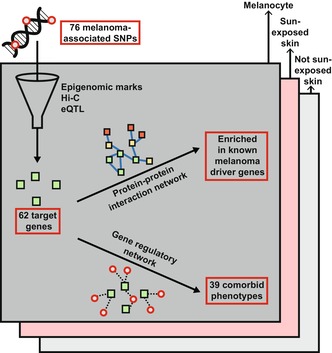

**Abbreviations**
CGC: cancer gene census
DR: depletion rank
eQTL: expression quantitative trait loci
GRN: gene regulatory network
GWAS: genome‐wide association study
LD: linkage disequilibrium
LOEUF: loss‐of‐function observed/expected upper bound fraction
LoF: loss‐of‐function
MPRA: massively parallel reporter assay
PPIN: protein–protein interaction network
SNP: single nucleotide polymorphism
WT1: wilms' tumor 1

## 1 Introduction

Cutaneous melanoma is the deadliest form of skin cancer, with increasing worldwide incidence [1]. Understanding the underlying biological mechanisms driving melanoma, particularly individual risk and how it arises and develops, is crucial for better treatment and prevention. To this end, genome‐wide association studies (GWAS) have identified many genomic risk loci (each containing variants in linkage disequilibrium; LD) that are associated with risk of developing melanoma [2]. However, GWAS associations are statistical in nature and thus do not inform on how associated risk loci contribute to the biological mechanisms underlying melanoma.

Most (> 90%) single nucleotide polymorphisms (SNPs) identified by GWAS lie in the non‐coding regions of the genome [3]. As such, one possible mechanism by which these SNPs affect disease risk is through altering regulatory processes that modify the transcription level of distal target genes. Identifying which potential regulatory sites are altered can be achieved by overlapping GWAS loci with maps of regulatory elements (i.e., Roadmap Epigenomics [4] and ENCODE [5]) that define histone marks and open chromatin regions across diverse cell types.

Regulatory sites (e.g., enhancers) act on target genes from a distance, meaning that identifying the target gene for a putative regulatory element requires more than just understanding which genes are nearby. Thus, methods that integrate data on 3D genome organization (e.g., Hi‐C [6]) can greatly improve predictions of which distal genes are regulated by a particular enhancer element. Furthermore, methods which directly associate loci to regulation of transcription (e.g., in the form of eQTLs) can also be used to find enhancer gene associations. Integrating these distinct layers of biological information is instrumental for the functional annotation of GWAS loci [7]. However, as gene regulatory landscapes vary cell‐to‐cell [8], such integrative analyses should ideally be performed using data from tissues and cell types that are relevant to the condition of interest (e.g., skin and its subtypes for melanoma).

Beyond simply identifying gene targets for SNPs, proteins encoded by the affected genes are themselves targets of larger co‐regulatory mechanisms via protein–protein interaction network (PPIN) [9]. Therefore, it is important to consider the context of gene target encoded proteins within this PPIN [10, 11]. Such a network‐based analysis may reveal biologically relevant patterns that extend beyond the limited information on individual genes.

This study aimed to gain novel insights into the mechanisms underlying melanoma risk by integrating information on melanoma GWAS risk loci with epigenomic markers, 3D genome organization, and eQTL information from melanoma‐relevant tissues and cell types. We found upregulated and downregulated target genes, both near and far away (> 1 Mb in the linear sequence) from the regulatory risk loci, with known and novel roles in the etiology of melanoma. Through PPIN, we revealed connections between these target genes which were enriched for known melanoma driver genes. We then integrated these target genes into gene regulatory network (GRN) to reveal patterns of gene regulation that connect melanoma to its comorbidities (e.g., skin pigmentation, immune response, lipid levels, and other cancers). This study provides novel insights into the biological implications of genetic variants associated with melanoma risk and provides a starting point for further experimental validation.

## 2 Materials and methods

### 2.1 Identification and fine‐mapping of melanoma risk loci

Single nucleotide polymorphisms (SNPs) associated with cutaneous melanoma were retrieved from the most recent GWAS meta‐analysis [2] through GWAS catalog [12] (study accession GCST010304; on February 2022). This included 68 genome‐wide significant SNPs (*P* ≤ 5 × 10^−8^) and 8 SNPs reaching suggestive significance (*P* ≤ 5 × 10^−6^). In total, 76 independent lead SNPs (representing 76 melanoma risk loci) were retrieved for use in subsequent analyses. To obtain the SNPs tagged by each of the locus, the European population of the 1000 Genomes Phase 3 data [13] was used to select variants that are in high LD (*r* ^2^ ≥ 0.8) with each of the original 76 lead SNPs (*n* = 2137).

To prioritize potentially functional regulatory SNPs in each locus, we identified the SNPs that are located in putative active enhancers/promoters/open chromatin regions in melanocyte or melanoma using one or more of the following criteria:

1. Variant is located within a human melanocyte H3K27ac AND H3K4me1 ChIP‐seq peak
2. Variant is located within a human melanocyte H3K27ac AND H3K4me3 ChIP‐seq peak
3. Variant is located within a human melanocyte DHS peak
4. Variant is located within a melanoma cell line DHS peak
5. Variant is located within a melanoma short‐term culture FAIRE‐seq peak
6. Variant is implicated as a significant massively parallel reporter assay (MPRA) variant

Melanocyte ChIP‐seq peaks were obtained from human foreskin melanocyte cultures from two individuals available from Roadmap Epigenomics [4]. Melanocyte DHS peaks were obtained from human foreskin and epidermal melanocyte cultures from three individuals available from Roadmap Epigenomics and ENCODE [5]. Melanoma DHS peaks were obtained from two melanoma cell line (SK‐MEL‐5 and RPMI9751) cultures available from ENCODE. Melanoma FAIRE‐seq peaks were obtained from 11 melanoma culture samples from Verfaillie et al. [14]. MPRA significant variants (*n* = 39) were obtained from Choi et al. [15]. Where appropriate, LiftOver tool [16] was used to convert coordinates of genomic features from genome build hg19 to hg38.

### 2.2 Sequence constraint analysis

Depletion rank (DR) score data were downloaded from Halldorsson et al. [17] (available as a supplementary data). The file contains the associated DR score for each overlapping set of 500‐bp windows in the genome with a 50‐bp step size. To obtain raw DR scores, the python library pybedtools [18] was used to identify every 500‐bp window and its associated DR score that overlaps with each SNP in melanoma risk loci. The mean DR score for each SNP was then calculated and used for statistical testing using Kruskal–Wallis test followed by Dunn's *post‐hoc* test with Bonferroni correction for multiple comparisons.

### 2.3 Identification of target genes and determination of their effect direction

The CoDeS3D algorithm [19] was used to identify prioritized SNPs that acted as cell type and/or tissue‐specific spatial regulator of target gene expression. First, the Hi‐C chromatin contact data from human epidermal keratinocyte (NHEK) [20] and two ENCODE melanoma (SK‐MEL‐5 and RPMI9751) cell lines were used to find distal DNA fragments that spatially connect to each SNP. If a distal fragment overlapped an annotated gene coding region (based on GRCh38 gene annotation from GENCODE v26 [21]), then a spatial SNP‐gene pair was established. Only SNP‐gene pairs confirmed in at least two replicates in the Hi‐C data were tested for eQTL association. For each spatial SNP‐gene pair, the eQTL data from human melanocyte [22] and the two GTEx skin tissues (skin sun exposed lower leg & skin not sun exposed suprapubic) [23] were used to identify whether the SNP also associates with expression changes of the gene to establish a spatially‐constrained eQTL (“eQTL”) association with the target gene. Finally, the Benjamini–Hochberg False Discovery Rate (FDR ≤ 0.05) was used to adjust the eQTL‐target gene association *P*‐values.

Hierarchical bi‐clustering of the magnitude and effect direction (beta value) of each risk locus‐target gene pair was performed using the pheatmap [24] (v1.0.12) R package. eQTL with the lowest adj. *P*‐value in each locus was used. However, CoDeS3D by default reports the beta value direction based on the alternate allele of SNPs in dbSNP151, which might or might not be the risk allele. To accurately report the gene regulatory direction (up‐ or downregulation) relative to the risk allele of each locus – target gene pair, if the alternate allele was not the risk allele, the beta value direction outputted by CoDeS3D was switched (i.e., −1 to +1 and vice versa). If the eQTL with the lowest adj. *P*‐value in a locus was in LD with the lead SNP (but not the lead SNP itself), the risk allele was determined by identifying the allele with the highest LD with the risk allele of the lead SNP using LDlink [25]. Loci with unknown risk allele were excluded from this analysis.

### 2.4 Positional annotation of eQTLs

The r package haplor [26] (v4.0.2) was used to determine the functional class of each eQTL. haplor queries the web‐based tool haploreg (v4.2), which relies on the dbSNP functional annotation.

### 2.5 Identification of genes previously linked to melanoma

Genes previously linked to melanoma were identified through DisGeNET [27] (“Curated gene‐disease associations” file downloaded on May 2022 with diseaseType = disease, diseaseSemanticType = Neoplastic Process, diseaseName = melanoma), Melanoma Gene Database [28] (available as supplementary table 1 on the original publication), and GWAS catalog [12] (Trait label = melanoma, EFO ID = EFO_0000756, column = Mapped gene, accessed May 2022).

### 2.6 Loss‐of‐function (LoF) tolerance analysis

The tolerance for LoF mutations was assessed using the “Loss‐of‐Function Observed/Expected Upper Bound Fraction” (LOEUF) metric, which was obtained from the gnomAD database (v2.1.1) [29].

### 2.7 Functional and disease enrichment analysis

Functional enrichment analysis of the target genes was conducted using g:Profiler [30] (September 2022). Disease enrichment analysis was performed using David Bioinformatics Resources [31] (September 2022) using the information from DisGeNET. Adjusted *P*‐value ≤ 0.05 was set as the cut‐off criteria.

### 2.8 Drug interaction analysis

The Drug Gene Interaction database 4.0 [32] (DGIdb, http://dgidb.org) was interrogated for information on drugs that target the products of the target genes.

### 2.9 Protein–protein interaction network (PPIN) analysis

Protein–protein interaction data were obtained from STRING (v11.5) [9] and included the following parameters: experiments, text‐mining, databases, co‐expression, neighborhood, gene fusion, and co‐occurrence, species limited to “Homo sapiens”, and interaction score > 0.9. We also obtained protein–protein interaction data from HumanNet‐XC [33] (v3) database.

Tissue‐specific PPINs were constructed by first taking the eQTL target genes identified in the tissue of interest (e.g., the target genes identified by CoDeS3D in melanocyte tissue for melanocyte PPIN) and parsing it to STRING/HumanNet to identify every interacting protein up to 4 edges away. The input gene list was assigned as the level 0 protein set. Next, proteins that directly interact with any level 0 protein but themselves are not part of the level 0 protein set are assigned level 1. Proteins that directly interact with any level 1 protein but themselves are not part of the level 0 nor 1 protein set are assigned level 2, and so on. This generated level 0–4 protein set for each tissue. Hypergeometric test was used to test for enrichment of melanoma driver genes in each of the PPIN level in each tissue. Melanoma driver genes (*n* = 64) were retrieved from the Cancer Gene Census database [34] (July 2022). cytoscape (v3.9.1) [35] was used to visualize the protein interaction pathways from the level 0 protein set to proteins encoded by melanoma driver genes at level 1–2. Interactions that did not lead to a connection between level 0 protein and driver gene at level 1–2 (e.g., the interaction between one level 1 protein and another level 1 protein) were not shown.

### 2.10 Comorbidity analysis

Tissue‐specific gene regulatory networks (GRNs) were constructed by calling all spatially constrained eQTLs (“eQTLs”) involving all common SNPs (MAF ≥ 0.05) in melanocyte, sun‐exposed skin, and not sun‐exposed skin tissue overlapping a melanoma‐relevant genomic signature via the CoDeS3D pipeline (same criteria as the original melanoma spatial eQTL calling, but on all SNPs instead of just melanoma risk SNPs). Each GRN was then queried to identify all eQTLs targeting melanoma target genes found originally in that tissue (i.e., the 62, 79, and 78 melanoma target genes found originally in melanocyte, sun‐exposed skin, and not sun‐exposed skin, respectively) or the 248 DisGeNET melanoma genes as comparison. For each GRN tissue, the identified eQTLs were tested to identify every trait in GWAS catalog whose SNPs overlap with any of the identified eQTLs within LD *r* ^2^ ≥ 0.8. Curated GWAS associations were downloaded from the NHGRI‐EBI GWAS Catalog [12] on 06‐11‐2022. For each trait, statistically significant eQTL enrichments were determined by hypergeometric test (*P* ≤ 0.05). The Benjamini–Hochberg False Discovery Rate (FDR ≤ 0.05) was used to adjust the *P*‐values. Additionally, bootstrapping (*n* = 350) was performed by randomizing the input gene set equal to the size of the original melanoma target genes found in each tissue (or the 248 DisGeNET melanoma genes). For each trait, bootstrapping *P*‐value was determined using the formula *P* = (no. of simulation for which the trait in question is significant after hypergeometric test + 1)/(350 + 1) as previously recommended [36]. Traits with hypergeometric adj. *P*‐value and bootstrapping *P*‐value ≤ 0.05 were deemed to be significantly associated with melanoma. Hierarchical clustering was performed using the pheatmap (v1.0.12) R package on the log number of GWAS SNP of each significant trait that overlap at least one eQTL targeting a melanoma target gene (within LD *r* ^2^ ≥ 0.8) + 1.

## 3 Results

### 3.1 Fine‐mapping identifies risk SNPs in melanoma GWAS loci under stronger purifying selection

**Fig. 1:**
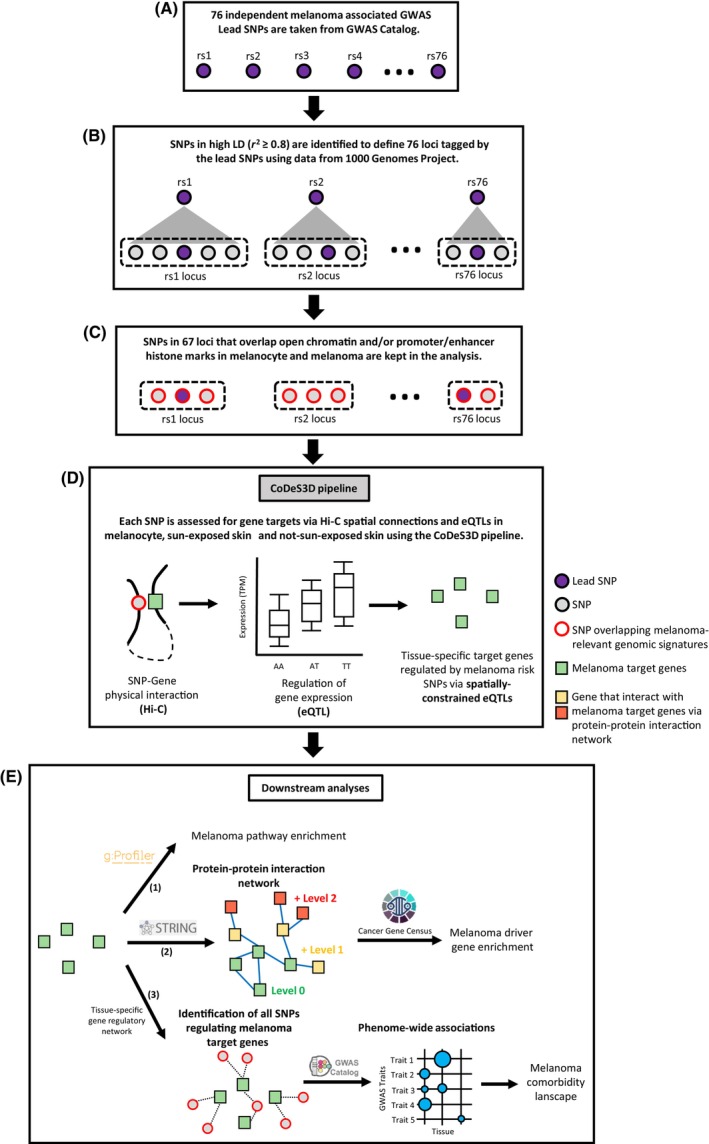
Overview of the analytical approach used in this study. (A) Seventy‐six independent melanoma‐associated meta‐GWAS lead SNPs were obtained. (B) SNPs in high (*r* ^2^ ≥ 0.8) linkage disequilibrium (LD) with the 76 lead SNPs were identified to define 76 melanoma risk loci. (C) SNPs in each locus that overlap melanoma‐relevant regulatory genomic signatures were prioritized and retained in the analysis. (D) The CoDeS3D pipeline was used to identify the tissue‐specific target genes of the prioritized SNPs. (E) Additional downstream analyses of the target genes consisted of three parts: (1) Functional profiling; (2) Construction of tissue‐specific protein–protein interaction networks to identify interacting proteins, which were tested for enrichment with known melanoma driver genes; (3) Construction of tissue‐specific gene regulatory network maps that were used to identify enrichment of traits as a proxy for genetic overlap/multimorbidity.

Meta‐analysis of GWAS for melanoma identified 76 independent lead SNPs associated with cutaneous melanoma risk [2]. However, these lead SNPs are not necessarily causal for melanoma as GWAS associations can result from various indirect mechanisms (e.g., effects from SNPs in linkage disequilibrium; LD) [37, 38]. Therefore, to increase the chance of capturing the biologically relevant signals for the association, melanoma risk loci were defined as the full set of SNPs in high LD (*r* ^2^ ≥ 0.8) with each of the 76 lead SNPs (*n* = 2137 SNPs in 76 loci) (Fig. 1A,B, Table S1). To prioritize SNPs within these 76 loci that are most likely to alter regulatory mechanisms, only those that overlap a melanoma‐relevant genomic signature (i.e., open chromatin regions, regions marked with promoter/enhancer histone marks in primary melanocytes and/or melanoma short‐term cultures, or SNPs that have been previously implicated as allele‐specific enhancers by massively parallel reporter assays (MPRA)) were retained for use in subsequent analyses (Fig. 1C). This resulted in 1094 prioritized SNPs that are spread across 67 loci (Table S2).

**Fig. 2:**
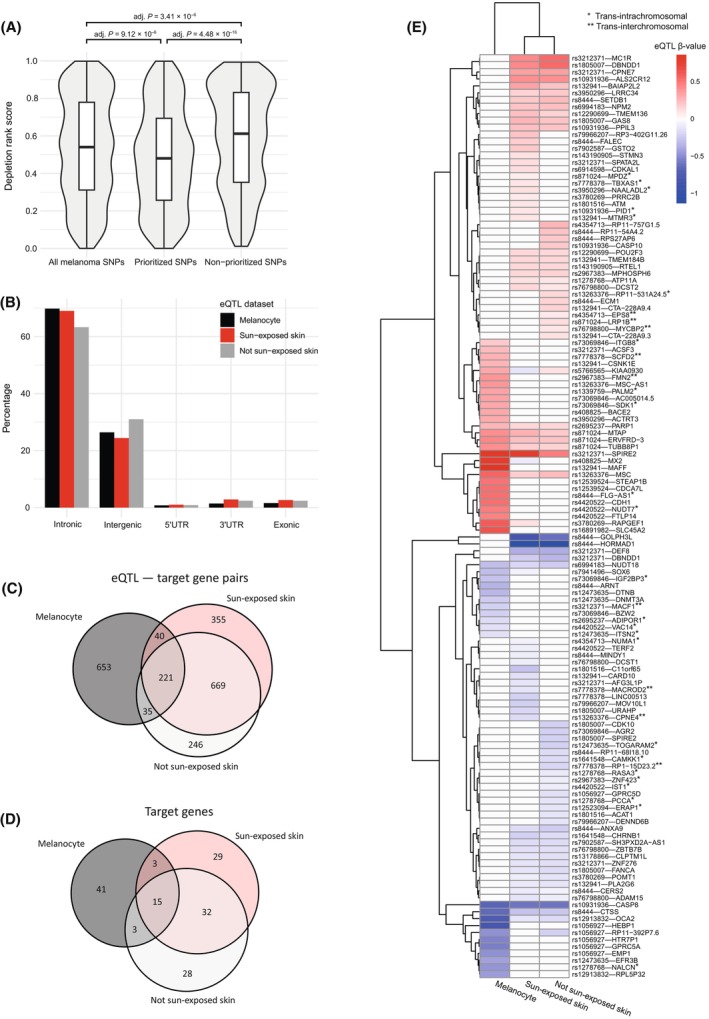
SNPs located within melanoma risk loci are associated with increased or decreased expression of 151 target genes in three melanoma‐relevant tissues. (A) SNPs in melanoma risk loci that overlap melanoma‐relevant genomic signatures are subject to increased purifying selection—measured by depletion rank score— compared to those that do not. Statistical significance was assessed using Kruskal–Wallis test followed by Dunn's *post‐hoc* test with Bonferroni correction for multiple comparisons. (B) The vast majority of melanoma‐associated eQTLs in melanocyte (98.4%), sun‐exposed skin (97.4%) and not sun‐exposed skin (97.6%) are located within intronic or intergenic genomic regions. There is minimal (C) eQTL‐gene pairs and (D) target genes overlap within the three melanoma‐relevant tissues. (E) The magnitude and effect direction of genes targeted by each risk locus varies. Red and blue color indicate association with up‐ and downregulation, respectively. * and ** indicate *trans‐intrachromosomal* and *trans‐interchromosomal* regulation, respectively.

To test if the prioritized SNPs were under purifying selection (a hallmark of active regulatory sequences [39]), we compared the sequence constraints associated with the 1094 prioritized SNPs and the 1043 SNPs that do not overlap melanoma‐relevant genomic signatures. For this analysis, we used the depletion rank (DR) score, which is a recently developed measure of genome‐wide sequence constraint [17]. First, the mean DR scores associated with each SNP were calculated (Table S3). Next, the difference in the distribution of mean DR scores between each SNP group was tested using Kruskal–Wallis test followed by Dunn's *post‐hoc* test with Bonferroni correction for multiple comparisons. The result identified the prioritized SNPs as being in genomic regions with significantly lower DR score compared to the non‐prioritized SNPs (Fig. 2A, Fig. S1). This is consistent with the melanoma prioritized SNPs being in genomic regions with stronger purifying selection and supports their potential functional importance.

### 3.2 SNPs in melanoma risk loci alter regulatory regions that target 151 candidate genes

Putative gene targets for the 1094 prioritized SNPs were identified using Hi‐C spatial data generated from normal human epidermal keratinocytes (NHEK) and two melanoma (SK‐MEL‐5 and RPMI79510) cell lines, and eQTL data from melanocyte, sun‐exposed skin (lower leg), and not sun‐exposed skin (suprapubic) through the CoDeS3D pipeline [19] (Fig. 1D). For each eQTL dataset, only SNP‐gene connections confirmed in at least two replicates in the Hi‐C data were tested for eQTL association(s).

Separating the analysis according to the source eQTL datasets resulted in the identification of 635 significant (FDR‐adjusted *P* ≤ 0.05) spatially‐constrained eQTLs (hereafter “eQTLs”) associated with the expression of 62 target genes in melanocytes; 488 eQTLs associated with 79 target genes in sun‐exposed skin; and 454 eQTLs associated with 78 target genes in not‐sun‐exposed skin (Table S4a–c). Combining the three datasets identified 151 genes that were targeted by eQTLs within 42 loci. In each tissue, the majority of SNPs forming these eQTLs were located in the non‐coding regions of the genome: either intronic or intergenic (Fig. 2B, Table S5a–c).

Comparisons identified minimal overlap between unique eQTL‐target gene pairs identified within the melanocyte and the two skin tissues (13.5% overlap with sun‐exposed skin, 14% with not‐sun‐exposed skin) (Fig. 2C). Of note, despite the fact that the eQTL data from sun and not‐sun‐exposed skin was acquired from a cohort of individuals within the GTEx cohort [23], only 56.8% of the eQTL‐gene pairs were shared between these tissue samples, consistent with the exposure‐ and tissue‐dependence of these eQTLs [40] (Fig. 2C). Of the 151 target genes we identified, 15 genes were targeted by eQTLs in all three tissue types, 38 were targeted in two tissue types (with most overlaps between the sun‐ and not‐sun exposure skin samples), while 98 (65% of target genes) were specific to only one of the three tissue types (Fig. 2D).

For each gene, the direction of eQTL effect was determined by adjusting the beta value sign according to the risk allele identity of the GWAS‐associated locus (Table S6a–c). This adjustment enabled the identification of genes whose up‐ or downregulation correlates with melanoma risk. The eQTL effect directions were collinear for the majority of shared genes across tissues (Fig. 2E). However, the regulatory impacts of some eQTLs switched direction in the different tissues. For example, *MX2* were downregulated in sun‐exposed skin but upregulated in melanocyte (Fig. 2E).

Trait‐associated SNPs identified as eQTLs often do not target the nearest annotated genes [41]. Here, we found 110 of the 151 genes (73%) were regulated by variants ≤ 1 Mb away (*cis*), with the remaining 27% of genes regulated by variants located > 1 Mb away (*trans‐intrachromosomal*; *n* = 29) or on different chromosome (*trans‐interchromosomal*; *n* = 12) (Table S4a–c, Fig. 2E). Our results are consistent with loci associated with melanoma risk contributing to that risk by up‐ or downregulating proximal and distal target genes.

### 3.3 Genes targeted via long‐distance regulation are novel and intolerant to loss‐of‐function mutations

**Fig. 3:**
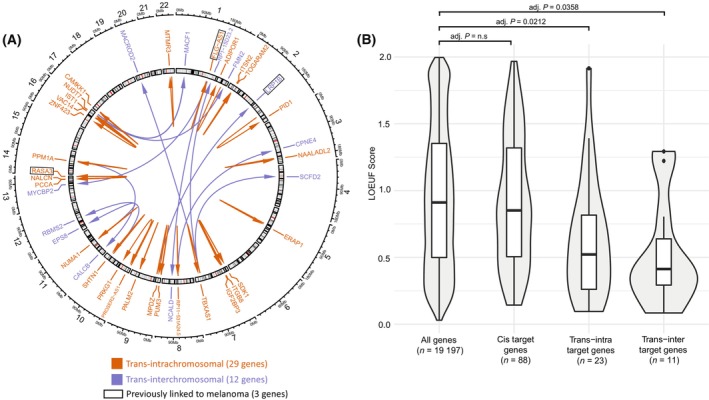
eQTLs located within melanoma risk loci are associated with the regulation of 42 genes in *trans*. (A) Circos plot visualizing the genome‐wide *trans* regulatory landscape of melanoma risk loci. (B) *Trans* regulated genes are more intolerant of loss‐of‐function mutations as estimated using LOEUF score. Statistical significance was assessed using Kruskal–Wallis test followed by Dunn's *post‐hoc* test with Bonferroni correction for multiple comparisons.

This study identified a total of 41 genes across the three tissues that were regulated in *trans* (29 *trans‐intrachromosomal* and 12 *trans‐interchromosomal*) by eQTLs located in 23 independent risk loci across 14 chromosomes (Fig. 3A). This represents the majority of loci that displayed eQTL activity (*n* = 23/42 eQTL containing loci), consistent with the interpretation that *trans* regulation is a common property of the regulatory elements associated with melanoma risk loci. Previous studies have demonstrated that *trans*‐eQTLs display greater tissue specificity than *cis*‐eQTLs [42, 43]. Consistent with this, all *trans* regulated genes found in this study were specific to only one of the three eQTL datasets (Table S4a–c, Fig. 2E).

Of the 151 target genes we identified, 48 genes (31.8%) have been previously linked to melanoma in either DisGeNET [27], Melanoma Gene Database (MGDB) [28] and/or GWAS Catalog [12] (Table S7). However, the vast majority (38 out of 41; 93%) of *trans*‐regulated genes are novel and have not been previously linked to melanoma (Fig. 3A, Table S7).

Genes that are developmentally essential are intolerant to loss‐of‐function (LoF) mutations [29]. In particular, cancer driver genes are more likely to be LoF intolerant [44]. Therefore, we hypothesized that the targets of the germline regulatory melanoma risk loci would be enriched for genes intolerant to LoF mutations. Stratifying the target genes according to their regulatory type identified *trans* regulated genes as having a markedly lower tolerance to LoF mutations when compared to *cis* regulated genes (Fig. 3B). Indeed, *cis* regulated genes alone were not statistically different (Kruskal–Wallis test followed by Dunn's *post‐hoc* test with Bonferroni correction for multiple comparisons) from the background dataset of all gnomAD genes (Fig. 3B, Table S8). Therefore, the *trans* regulated genes identified in this study represent potentially novel candidates that are intolerant to LoF mutations and thus are likely to be part of the gene set that drives melanomagenesis.

### 3.4 Gene‐set analysis identified enrichment of target genes in known melanoma pathways

Functional profiling of the 151 target genes (g:Profiler [30]; Table S9) identified an enrichment of genes important in the apoptosis pathway (i.e., *CASP8*, *PARP1*, *CASP10*, *CTSS*, *ATM*) and white hair phenotype (i.e., *MC1R*, *RTEL1*, *SLC45A2*, *OCA2*). These recapitulate the role of the regulation of cell senescence and pigmentation in the risk of developing melanoma [45, 46]. Additionally, the target genes were also enriched in the binding motif of *Wilms' tumor 1* (*WT1*), a developmental transcription factor commonly dysregulated in hematologic malignancies and solid tumors such as breast cancer, ovarian cancer and glioblastoma [47]. In melanoma, *WT1* expression was implicated as a differentiating marker between tumor and normal melanocytic nevi [48]. Our findings suggest a possible role of the *WT1* regulatory machinery in melanomagenesis. Of note, the enrichment of *WT1* motif was predominantly driven by the *trans*‐regulated genes (Table S9), consistent with their critical function during development, agreeing with their observed intolerance to LoF mutations.

We screened the Drug Gene Interaction database [32] (DGIdb 4.0) to identify target genes that encodes potentially targetable products. Out of the 151 target genes, 30 are targeted by at least one drug (Table S10). Notably, 15 (50%) of these genes have not been linked to melanoma before and 8 (26.7%) are regulated in *trans* (Fig. S3).

To further elucidate the potential roles of the target genes in known diseases, we performed a disease enrichment analysis using DAVID [31]. The analysis used information from DisGeNET [27] and identified known melanoma genes as being the most enriched within the target gene set (*n* = 11, adj. *P* = 3.58 × 10^−3^, Table S11). This melanoma gene set included *MTAP*, *SLC45A2*, *CASP8*, *CASP10*, *MC1R*, *SETDB1*, *ARNT*, *MX2*, *ATM*, and *PARP1*. We also identified *LRP1B*, which is a target of recurrent somatic mutations in melanoma [49], exemplifying a potential interplay between germline and somatic events in melanomagenesis.

### 3.5 Tissue‐specific target genes interact with a core set of melanoma driver genes through protein–protein interaction network

The Cancer Gene Census (CGC) contains a catalog of genes that have been causally implicated in cancer [34]. These causal genes include somatic driver genes and high‐penetrance germline genes that are responsible for inherited cancer syndromes. Due to their small effect sizes, cancer GWAS SNPs are recognized as having low‐penetrance. As such, these different types of genetic alterations are typically investigated separately. However, their combined analysis might reveal undiscovered interactions between them. Specifically, it is likely that the convergence between these different types of genetic alterations may occur at the level of protein–protein interactions. Therefore, we constructed protein–protein interaction networks (PPINs; using data from the STRING database [9] with the highest confidence score of > 0.9 as threshold) to identify the interactions between the melanoma target genes that we identified, and known melanoma somatic driver and highly‐penetrant germline genes (hereafter “driver genes”).

**Fig. 4:**
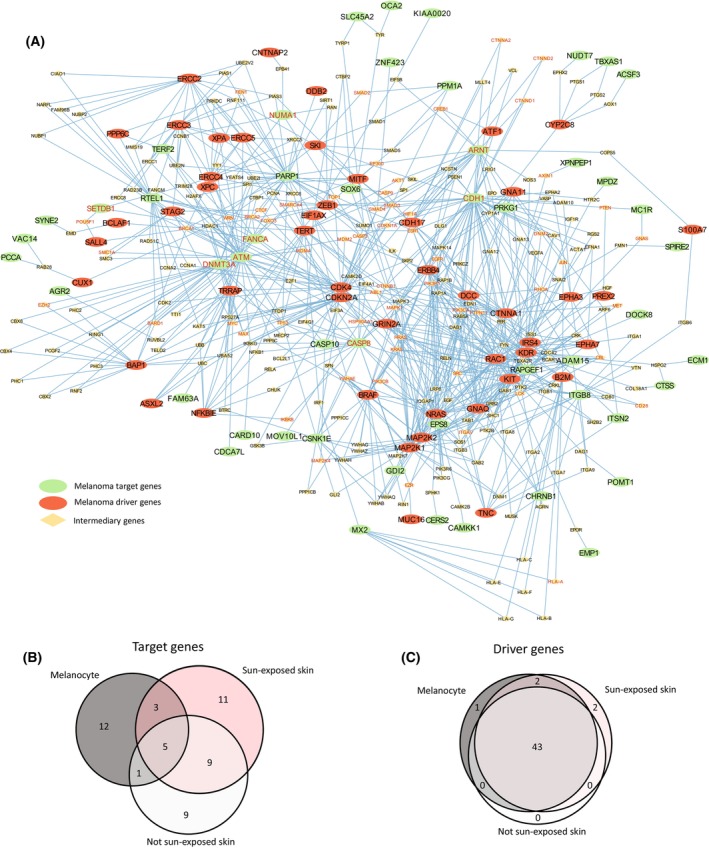
Proteins encoded by genes targeted by melanoma‐associated eQTLs interact with proteins encoded by melanoma driver genes within a functional protein–protein interaction network (PPIN). (A) Protein interaction pathways from melanoma target genes from all tissues at level 0, to melanoma driver genes at levels 1 and 2. Proteins encoded by melanoma target genes are colored green, while those encoded by known melanoma drivers are colored red. Red text indicates known cancer drivers that have not been annotated as melanoma drivers within the Cancer Gene Census database. The overlaps of (B) melanoma target genes and (C) melanoma driver genes within the tissue specific PPINs.

Briefly, PPINs were constructed using the proteins encoded by melanoma target genes as the level 0 protein set. The PPIN was then expanded to level 1–4, where level 1 proteins are one edge away from (directly connect to) any of the level 0 proteins, level 2 proteins are two edges away, and so on. This process was done separately using the protein products of melanocyte, sun‐exposed skin, and not sun‐exposed skin target genes as the starting (level 0) protein set (Tables S12 and S13a). This generated three PPINs that each identified the shortest connections between the tissue‐specific level 0 proteins and proteins at level 1–4. We subsequently performed an enrichment analysis to identify if the protein products of melanoma target genes were enriched for interactions with the protein products of known melanoma driver genes (*n* = 64; curated by the CGC [34]). There was significant enrichment of driver genes at level 1 and level 2 of the PPINs of all three tissues (Table S14a). Collectively, 50 of the level 0 proteins encoded by the target genes across the three tissues interact with 48 (of 64) proteins encoded by melanoma driver genes on levels 1 and 2 (Fig. 4A). This forms a single protein cluster (Fig. 4A) which is enriched in KEGG cancer pathways. While the target genes that are involved in these protein interactions are tissue‐specific (Fig. 4B), the majority of the driver genes (43 out of the 48, Fig. 4C) were shared between the tissue‐specific PPINs through distinct pathways, which include connections to *TERT*, *BRAF*, *NRAS*, *CDKN2A*, *MAP2K1*, and *MITF* (Fig. S2a–c). To further examine the findings from the STRING PPINs, we also analyzed the data using the HumanNet [33] database (Table S13b). Utilizing PPINs constructed from the HumanNet database, we identified enrichment at level 1; however, the interactions saturated quickly, precluding enrichment at level 2 (Table S14b). Notably, this analysis not only recovered all of the STRING identified driver genes at level 1 but also all of the target genes interacting with these driver genes (Fig. S4), highlighting a high degree of consensus between the two methods, with STRING exhibiting a more conservative approach overall.

### 3.6 Tissue‐specific target genes are involved in shared regulatory processes that link melanoma to other complex phenotypes

We hypothesized that a subset of melanoma target genes are pleiotropic and responsible for the association between melanoma and comorbid complex phenotypes. Prior studies that have attempted to elucidate comorbid phenotypes with melanoma have relied upon: (a) *a priori* selection of the interacting phenotypes; and (b) *a priori* selection of the potential pleiotropic genes underlying the associations [50, 51]. However, these prior assumptions limit the identification of both the interacting phenotypes and the pleiotropic genes driving the associations. Here, we performed a *de novo* analysis to elucidate the role of melanoma target genes in facilitating the connections between melanoma and other complex phenotypes.

We generated tissue‐specific gene regulatory networks (GRNs) comprised of gene targets whose expression levels are associated with all common SNPs (Table S15a–c). Each GRN (i.e., the melanocyte, sun‐exposed skin and not sun‐exposed skin GRN) was then queried to identify the set of eQTLs in the GRN which targeted the tissue‐specific melanoma target genes that were originally identified in that respective tissue (Table S4a–c). This step identified 1804 eQTLs that regulate melanoma target genes in the melanocyte GRN, 5056 eQTLs in the sun‐exposed skin GRN, and 4458 eQTLs in the not sun‐exposed skin GRN (Table S15a–c). These sets of eQTLs and their LD partners (*r* ^2^ ≥ 0.8) were then tested for enrichment for traits in the GWAS Catalog (hypergeometric test FDR ≤ 0.05 and bootstrapping *n* = 350) (Table S16a–c).

**Fig. 5:**
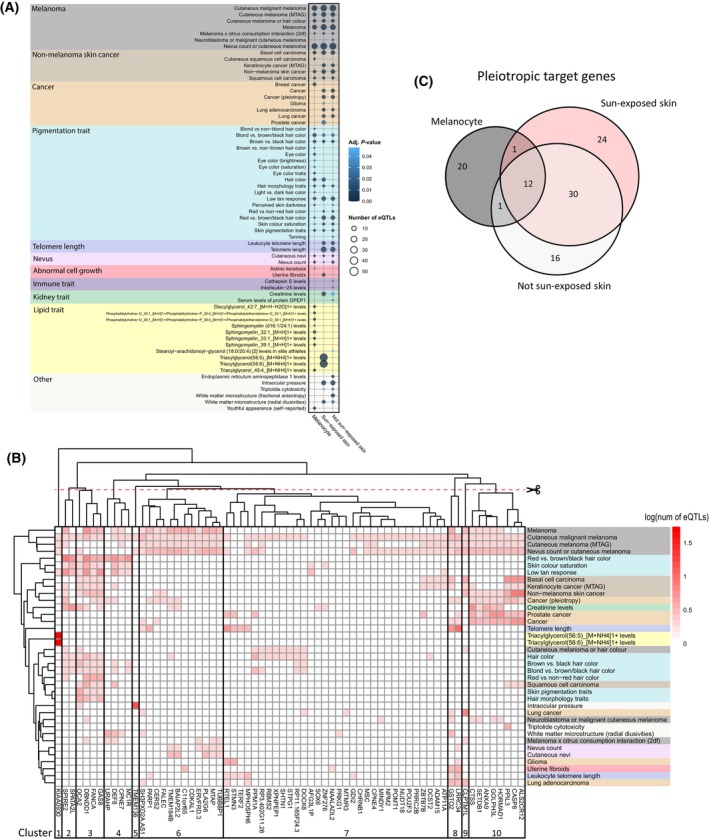
Comorbidity analysis was applied to melanocyte, sun‐exposed skin and not sun‐exposed skin gene regulatory network (GRN). (A) Significant melanoma‐associated traits in each of the three tissues (hypergeometric test adjusted and bootstrapping *P*‐value ≤ 0.05). (B) Hierarchical bi‐clustering identified the pleiotropic gene groups that were responsible the associations between melanoma and each significant trait in sun‐exposed skin GRN. (C) Overlap of the pleiotropic target genes that drive trait associations across the three tissues.

Across the three tissues, we identified 64 traits that were significantly enriched for these eQTLs (Fig. 5A). Screening the literature identified that these include: (a) traits with obvious relevance to melanoma (e.g., cancer, nevus count, pigmentation and telomere length traits); (b) traits that are supported by clinical and/or epidemiological observations (e.g., increased intraocular pressure in patients with iris melanoma [52], increased melanoma risk for patients with actinic keratosis [53]); and (c) traits that have not been (or are weakly) associated with melanoma (e.g., uterine fibroids and spontaneous coronary artery dissection). As a comparison, we repeated the analysis using DisGeNET melanoma‐associated genes as input (*n* = 248) (Table S17a–c). This revealed only 30 significant traits, with telomere length and nevus count traits notably absent (Fig. S5). Furthermore, the DisGeNET gene set identified just two pigmentation traits in melanocyte and failed to identify such associations in the other two tissues (Fig. S5). By contrast, our target genes identified 18 different pigmentation traits across the three tissues (Fig. 5A). Therefore, our approach potentially reveals a more detailed comorbidity landscape of melanoma.

Hierarchical bi‐clustering was next used to organize traits based on the number of GWAS SNPs of each trait that overlap GRN eQTLs regulating each target gene (*r* ^2^ ≥ 0.8, Table S17a–c). This elucidated unique patterns that show the contributions of specific target genes to the different traits. The pleiotropic target genes identified in sun‐exposed skin form 10 clusters (Fig. 5B). These clusters highlighted genes responsible for the association between melanoma and specific group(s) of traits. For example, genes in cluster 2 (*SPIRE2*, *SPATA2L*), 3 (*OCA2*, *DBNDD1*, *FANCA*, *GAS8*) and 4 (*URAHP*, *DEF8*, *CPNE7*, *MC1R*) underlie the associations between melanoma and pigmentation traits. Genes in cluster 8 (*GSTO2*, *LRRC34*), 9 (*CLPTM1L*) and 10 (*CTSS*, *SETDB1*, *ANXA9*, *GOLPH3L*, *HORMAD1*, *PPIL3*, *CASP9*, *ALS2CR12*) underlie the association between melanoma and cancers. Unlike other cancer types, melanoma and skin cancers were found to be uniquely associated with many of the pigmentation‐associated genes in clusters 2–5, in addition to the cancer associated genes in clusters 8–10. Additionally, we observed a unique association between melanoma and nevus count traits that were not identified as overlapping other cancer types, including skin cancers. Indeed, all genes associated with nevus count or cutaneous nevi in cluster 6 (*MTAP*, *TUBBP1*, *PLA2G6*, *ERVFRD.3*, *TMEM184B*, *BAIAP2L2*) were only associated with melanoma. Interestingly, these patterns were also seen in not sun‐exposed skin (Fig. S6a) and melanocyte (Fig. S6b). Therefore, our unbiased approach was able to capture the known relationship between UV damage and skin cancers that are not seen in other cancer types, and implicate the divergence of melanoma and other skin cancers due to the unique risk associated with nevus count traits.

The comorbidity landscape of melanoma across the three tissues was facilitated by pleiotropic target genes that are tissue‐specific (Fig. 5C). Nonetheless, the phenotypes identified as being comorbid in all three tissues largely agree with each other (e.g., traits relating to cancer, pigmentation traits, telomere length, and nevus) (Fig. 5A). For example, of the 16 total genes driving the association between melanoma and nevus count, only two (*MTAP* and *ERVFRD‐3*) were shared by all three tissues, and six (*MAFF*, *HEBP1*, *HTR7P1*, *EMP1*, *GPRC5A*, *C9orf66*) were specific to melanocyte (Table S18a–c).

## 4 Discussion

Alterations to the expression of distant genes may underlie the observed statistical association between certain SNPs and the risk of developing melanoma. This study integrated tissue‐specific data on epigenomic signals, chromatin interaction (Hi‐C) and gene expression changes (eQTL) to perform functional annotation of SNPs in melanoma GWAS loci. We identified 151 genes whose expression levels are altered (either up‐ or downregulated) by SNP risk alleles across 42 melanoma‐associated risk loci in melanocyte, sun‐exposed skin, and not‐sun‐exposed skin.

Among the genes we identified, many are known melanoma susceptibility genes including *CASP8*, *ARNT*, and *OCA2* (downregulated), *PARP1*, *SETDB1*, *MTAP*, *SLC45A2*, and *MC1R* (upregulated), and *MX2* (both up‐ and downregulated). For example, downregulation of *ARNT* has been demonstrated to confer melanoma metastatic potential through increasing ROS level [54]. Additionally, upregulation of *PARP1* has been shown to increase melanoma cell proliferation [55]. The regulatory impacts of some eQTLs switched direction in the different tissues. For example, *MX2* were downregulated in sun‐exposed skin but upregulated in melanocyte (Fig. 2E). Consistent with our result in melanocytes, a previous study has implicated rs398206 as a functional SNP in the rs408825 locus (21q22.3) that upregulates *MX2* via allelic binding of YY1 to its A risk allele [15]. Interestingly, we identified the same SNP as an eQTL that downregulates *MX2* in sun‐exposed skin, consistent with another study that suggested a tumor‐supressive feature of *MX2* where its downregulation was associated with melanoma disease progression [56]. This suggest that the contributions a subset of the germline inherited variants make to melanoma risk are mediated through complex regulatory mechanisms that are both cell type and context dependent, as may be reflected by the effect directions of their eQTLs.

Long‐distance (*trans*) regulation involving disease associated eQTLs remains understudied. The CoDeS3D pipeline searches for eQTL associations among variants with confirmed physical (Hi‐C) interactions, thus reducing the search space for *trans* associations, and improving the power to detect them. These *trans* regulated genes are enriched for the *WT1* motif (Table S9) and less tolerant to LoF mutations (Fig. 4B), demonstrating that the information they encode is developmentally critical. We have previously shown that genes regulated in *trans* are more likely to be intolerant to LoF mutations than *cis* regulated genes [57]. Most of the *trans*‐regulated genes we identified have not been previously linked to melanoma. However, *LRP1B* (regulated in *trans* by rs10811592 within the rs871024 locus; 9p21.3, in not sun‐exposed skin) was previously linked to melanoma through observations of recurrent somatic mutations within the gene [49]. Somatic disruption of the *LRP1B* gene has also been observed in several other cancer types, including lung [58], gastric [59], and squamous cancers [60, 61, 62]. Additionally, germline SNPs at the introns of *LRP1B* were recently associated with uveal melanoma [63]. These examples exemplify the connection between germline SNP predisposition and somatic gene mutational processes in melanomagenesis. Multiple SNPs in this locus were also associated with *cis* regulatory activity of *MTAP*, a well‐known melanoma susceptibility gene. As such, the *trans‐interchromosomal* regulation of *LRP1B* that involves this locus might constitute a secondary causal effect in addition to the *cis* regulation of *MTAP*. Our findings, together with previous reports that *trans*‐eQTLs display greater tissue specificity [42, 43], are enriched among disease loci [64], and explain significant proportions of gene expression heritability [65], highlight the critical contributions of long‐distance regulations to important molecular phenotypes, including melanoma risk. We contend that future studies would benefit by considering these new‐found associations that we identify as likely important for the development of melanoma.

Melanoma is a systems‐level network phenomenon, where germline susceptibility genes may impose risk, in part, by affecting driver genes through specific functional association pathways. Thus, linking germline to somatic processes might involve risk SNPs causing the development of a “fragile” phenotype in at‐risk individuals. The fragile phenotype could be impacted further by an environmental mutagenesis event (e.g., due to UV exposure) targeting the driver genes in question. Our protein–protein interaction networks demonstrated one potential mechanism for this in melanoma. Indeed, we found that at the protein level, melanoma target genes are enriched to interact with known melanoma driver genes 1–2 edges away, forming a single protein cluster enriched that is enriched cancer pathways (Fig. 3C). This new understanding of the specific interaction pathways between proteins encoded by melanoma target and driver genes opens a new avenue for the identification novel therapeutic targets.

The datasets we used were both a strength and weakness of this study. A primary strength lies in the integration of independent datasets across multiple biological levels that are specifically chosen for their relevance to melanoma. Yet, an inherent limitation persists: none of the datasets perfectly represent the true melanoma state. For instance, eQTL data sourced from bulk skin samples may inadvertently obscure melanocyte‐specific signals. While the melanocyte eQTL data aims to bridge this gap, its exclusive derivation from newborn males—lacking environmental exposure—potentially omits gene regulatory effects arising from specific environmental stimuli, such as sun exposure. It is thus expected that this context diversity impacts the results. This is illustrated by the differences we observed between the two skin samples. Nonetheless, extending beyond the limited information on individual genes showed that these tissue‐specific target genes converge on a core set of melanoma driver genes and associate with many common comorbid phenotypes. Thus, while the target genes of melanoma risk loci found in the three tissues are specific, they show higher‐level convergence at the phenotype and pathway level. It is possible that using eQTL data from malignant melanoma cells would provide further insights. However, such tumor tissues carry a high burden of somatic aberrations and therefore may not capture the underlying biology associated with inherited/germline melanoma risk.

## 5 Conclusions

In conclusion, by integrating tissue and cell type‐specific data on epigenomic signals, chromatin interaction and gene regulatory changes, we identified genes that are regulated by genetic variations within melanoma‐associated risk loci. Many of these genes have not been linked to melanoma before, with a substantial proportion of these being regulated in *trans*, mutationally constrained, potentially targetable by drugs, and identified previously in a somatic mutational role in melanoma or other cancer types. We identified that a subset of these target genes are involved in a shared regulatory processes that link melanoma to comorbid phenotypes and elucidated the contribution(s) of these pleiotropic genes to phenotype groupings across the three tissues. Our study provides novel insights into the biological implications of genetic variants associated with melanoma and provides a starting point for further experimental validation.

## Conflict of interest

The authors declare no conflict of interest.

## Author contributions

MP contributed to conceptualization, performed analyses, data interpretation, and wrote the manuscript. WS and JMOS supervised MP, conceptualized, and co‐wrote the manuscript. EG and TF contributed to the processing of melanocyte genotype and RNA‐seq expression data and revisions of the manuscript. All authors contributed to the article and approved the submitted version.

### Peer review

The peer review history for this article is available at https://www.webofscience.com/api/gateway/wos/peer‐review/10.1002/1878‐0261.13599.

## Supporting information


**Fig. S1.** Depletion rank score distribution of SNPs in melanoma risk loci that overlap each inclusion criteria.

**Fig. S2.** The specific contribution tissue‐specific protein–protein interaction network (PPIN) as a subset of the combined PPIN.

**Fig. S3.** 30 melanoma target genes are druggable based on DGIdb analysis.

**Fig. S4.** Interaction between melanoma target genes and melanoma driver genes in HumanNet protein–protein interaction networks (PPINs).

**Fig. S5.** Significant melanoma‐associated traits found using DisGeNET melanoma genes.

**Fig. S6.** Hierarchical clustering shows the contribution of pleiotropic target genes to different associated phenotypes.

**Table S1.** SNPs in high linkage disequilibrium (*r* ^2^ ≥ 0.8) with the 76 lead SNPs were identified.
**Table S2.** SNPs that overlap melanoma relevant genomic signatures were prioritized.
**Table S3.** Depletion rank (DR) score of each 500‐bp genomic window overlapping each SNP.
**Table S4a.** Melanoma‐associated eQTL – gene interactions identified in melanocyte.
**Table S4b.** Melanoma‐associated eQTL – gene interactions identified in sun‐exposed skin.
**Table S4c.** Melanoma‐associated eQTL – gene interactions identified in not sun‐exposed skin.
**Table S5a.** Positional annotation of the 635 eQTLs identified in melanocyte.
**Table S5b.** Positional annotation of the 488 eQTLs identified in sun‐exposed skin.
**Table S5c.** Positional annotation of the 454 eQTLs identified in not sun‐exposed skin.
**Table S6a.** The corrected beta value direction for each locus – gene pair found in melanocyte.
**Table S6b.** The corrected beta value direction for each locus – gene pair found in sun‐exposed skin.
**Table S6c.** The corrected beta value direction for each locus – gene pair found in not sun‐exposed skin.
**Table S7.** 48 of the 151 target genes have been previously associated with melanoma in either DisGeNET, Melanoma Gene Database (MGDB) and/or GWAS Catalog.
**Table S8.** Target genes and their LOEUF score obtained from the gnomAD database.
**Table S9.** Functional profiling results of the target genes using g:Profiler.
**Table S10.** A list of drugs that interact with the target genes from Drug Gene Interaction database.
**Table S11.** Disease enrichment analysis results of the target genes using DAVID.
**Table S12.** Target genes raw protein–protein interaction networks from STRING.
**Table S13a.** Proteins at level 0–4 in STRING protein–protein interaction networks.
**Table S13b.** Proteins at level 0–4 in HumanNet protein–protein interaction networks.
**Table S14a.** Enrichment of melanoma driver genes within STRING protein–protein interaction networks.
**Table S14b.** Enrichment of melanoma driver genes within HumanNet protein–protein interaction networks.
**Table S15a.** Melanocyte gene regulatory network.
**Table S15b.** Sun‐exposed skin gene regulatory network.
**Table S15c.** Not sun‐exposed skin gene regulatory network.
**Table S16a.** Traits significantly associated with melanoma in melanocyte using melanocyte target genes as input.
**Table S16b.** Traits significantly associated with melanoma in sun‐exposed skin using sun‐exposed skin target genes as input.
**Table S16c.** Traits significantly associated with melanoma in not sun‐exposed skin using not sun‐exposed skin target genes as input.
**Table S17a.** Traits significantly associated with melanoma in melanocyte skin using DisGeNET melanoma‐associated genes as input.
**Table S17b.** Traits significantly associated with melanoma in sun‐exposed skin using DisGeNET melanoma‐associated genes as input.
**Table S17c.** Traits significantly associated with melanoma in not sun‐exposed skin using DisGeNET melanoma‐associated genes as input.
**Table S18a.** SNP – gene connection details of significant melanoma‐associated traits in melanocyte using melanocyte target genes as input.
**Table S18b.** SNP – gene connection details of significant melanoma‐associated traits in sun‐exposed skin using sun‐exposed skin target genes as input.
**Table S18c.** SNP – gene connection details of significant melanoma‐associated traits in not sun‐exposed skin using not sun‐exposed skin target genes as input.

## Data Availability

All data generated during this study are included in this published article and its [Link], [Link], [Link], [Link], [Link], [Link], [Link] files. Access to melanocyte genotype and RNA‐seq expression data from 106 individuals was approved by the dbGaP (https://www.ncbi.nlm.nih.gov/gap/) Data Access Committee (Project ID: 30073, accession: phs001500.v1.p1), and processed according to the GTEx pipeline (https://github.com/broadinstitute/gtex‐pipeline) as previously described [66]. The CoDeS3D pipeline used to find target genes via spatially constrained eQTLs is available on github (https://github.com/Genome3d/codes3d‐v2). A version of the multimorbid3D [10] pipeline used in this study to identify level 0–4 protein set and comorbid traits is available on github (https://github.com/MichaelPudjihartono/multimorbid3D). Data analyses and visualizations were performed using python (version 3.8.12) through jupyter notebook (version 6.4.6) or using r (version 4.0.4) through rstudio (version 1.4.1106). Additional in‐house scripts used for data wrangling are available upon request.
