# Supplementary material for: Links between melanoma germline risk loci, driver genes and comorbidities: insight from a tissue‐specific multi‐omic analysis
Source: Mol Oncol. 2024 Feb 3;18(4):1031–48. doi: 10.1002/1878-0261.13599 (PMC10994230; doi:10.1002/1878-0261.13599)
Supplement: Supplementary file 1 — Fig. S1. Depletion rank score distribution of SNPs in melanoma risk loci that overlap each inclusion criteria. [file MOL2-18-1031-s006.pdf]

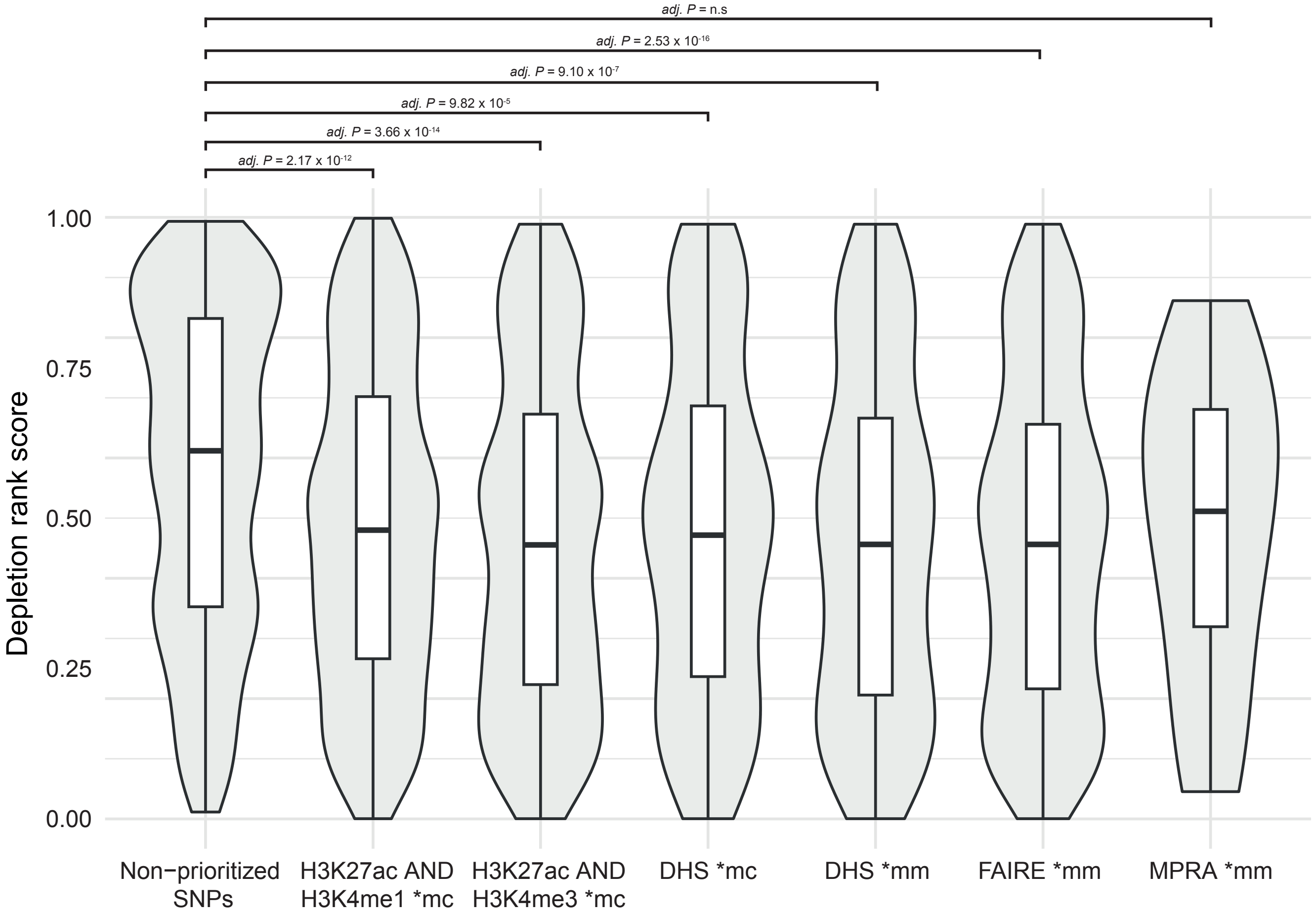

Supplementary Figure 1. Depletion rank score distribution of SNPs in melanoma risk loci that overlap each inclusion criteria. H3K27ac AND H3K4me1 indicate SNPs located within a H3K27ac and H3K4me1 ChIP-seq peak. H3K27ac AND H3K4me3 indicate SNPs located within a H3K27ac and H3K4me3 ChIP-seq peak. DHS indicate SNPs located within a DNAse hypersensitivity site (DNase-seq) peak. FAIRE indicate SNPs located within a FAIRE-seq peak. MPRA indicate SNPs significantly implicated as enhancer through MPRA. * Cell of origin are indicated, mc indicate data is from primary human melanocyte culture, mm indicate data is from melanoma cell line or short term culture. Statistical significance was assessed using Kruskal-Wallis test followed by Dunn's post-hoc test with Bonferroni correction for multiple comparisons.
