# Supplementary material for: Links between melanoma germline risk loci, driver genes and comorbidities: insight from a tissue‐specific multi‐omic analysis
Source: Mol Oncol. 2024 Feb 3;18(4):1031–48. doi: 10.1002/1878-0261.13599 (PMC10994230; doi:10.1002/1878-0261.13599)
Supplement: Supplementary file 2 — Fig. S2. The specific contribution tissue‐specific protein–protein interaction network (PPIN) as a subset of the combined PPIN. [file MOL2-18-1031-s005.pdf]

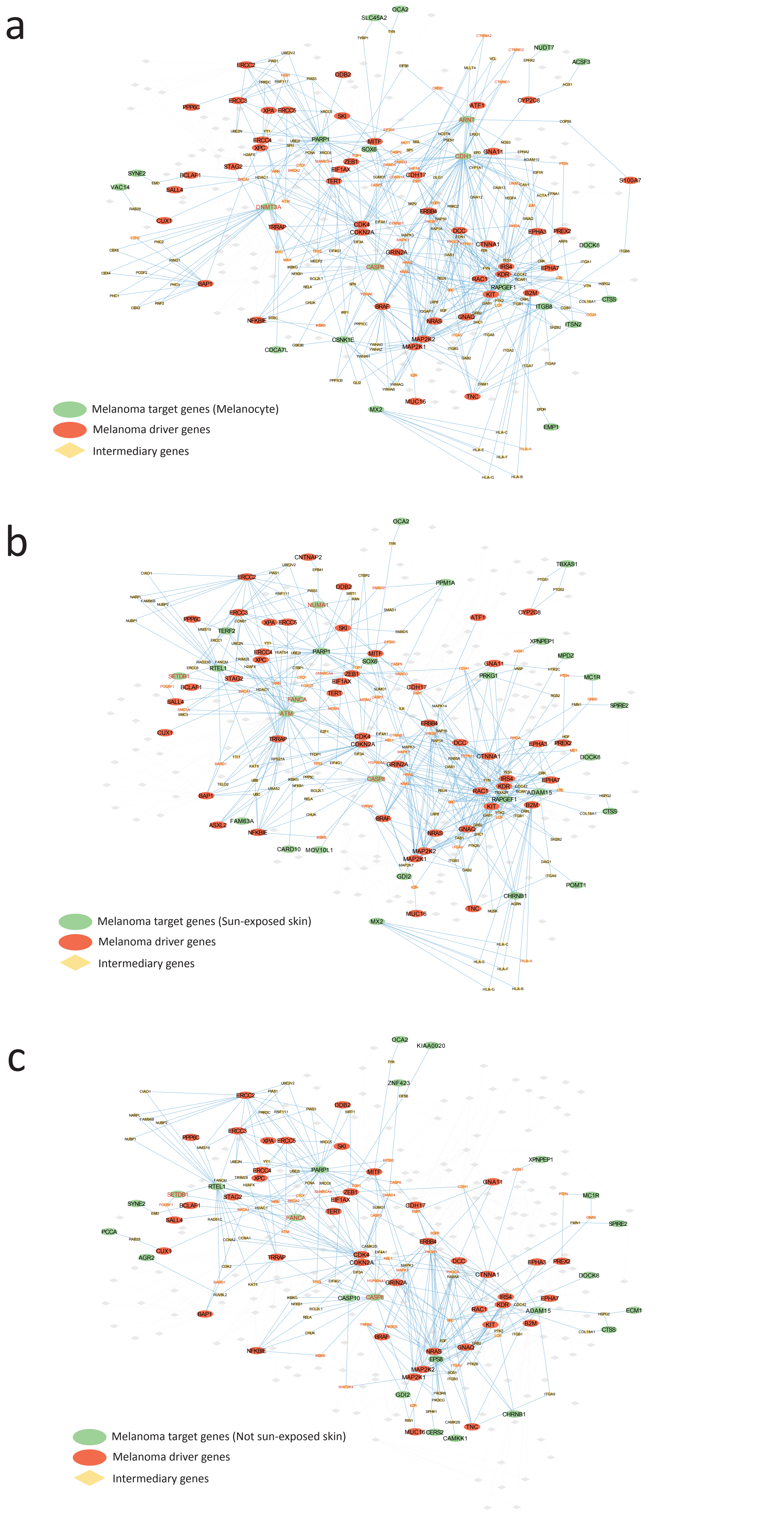

Supplementary Figure 2. The specific contribution tissue-specific protein-protein interaction network (PPIN) as a subset of the combined PPIN. STRING PPIN shows the interaction between protein products of melanoma target genes in (a) melanocyte, (b) sun-exposed skin, (c) not sun-exposed skin and known melanoma driver genes at level 1-2. Proteins encoded by melanoma target genes are coloured green, while those encoded by known melanoma drivers are coloured red. Red text indicate known cancer drivers that are not annotated as melanoma drivers in the Cancer Gene Census database.
