# Supplementary material for: Links between melanoma germline risk loci, driver genes and comorbidities: insight from a tissue‐specific multi‐omic analysis
Source: Mol Oncol. 2024 Feb 3;18(4):1031–48. doi: 10.1002/1878-0261.13599 (PMC10994230; doi:10.1002/1878-0261.13599)
Supplement: Supplementary file 3 — Fig. S3. 30 melanoma target genes are druggable based on DGIdb analysis. [file MOL2-18-1031-s002.pdf]

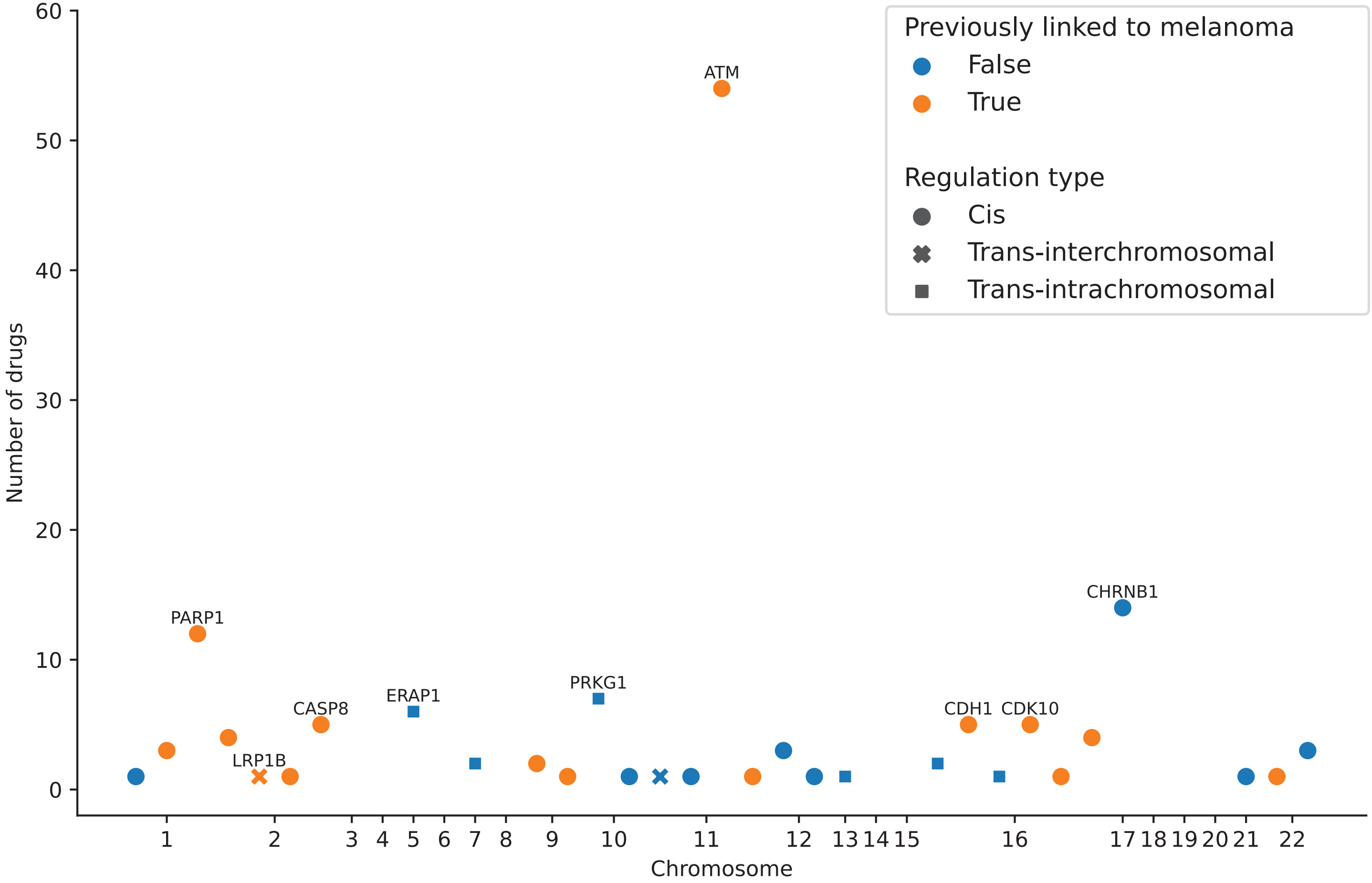

Supplementary Figure 3. 30 melanoma target genes are druggable based on DGIdb analysis. 50% (15) of these target genes have not been linked to melanoma before. 26.7% (8) of these target genes are regulated in *trans*.
