# Supplementary material for: Links between melanoma germline risk loci, driver genes and comorbidities: insight from a tissue‐specific multi‐omic analysis
Source: Mol Oncol. 2024 Feb 3;18(4):1031–48. doi: 10.1002/1878-0261.13599 (PMC10994230; doi:10.1002/1878-0261.13599)
Supplement: Supplementary file 4 — Fig. S4. Interaction between melanoma target genes and melanoma driver genes in HumanNet protein–protein interaction networks (PPINs). [file MOL2-18-1031-s003.pdf]

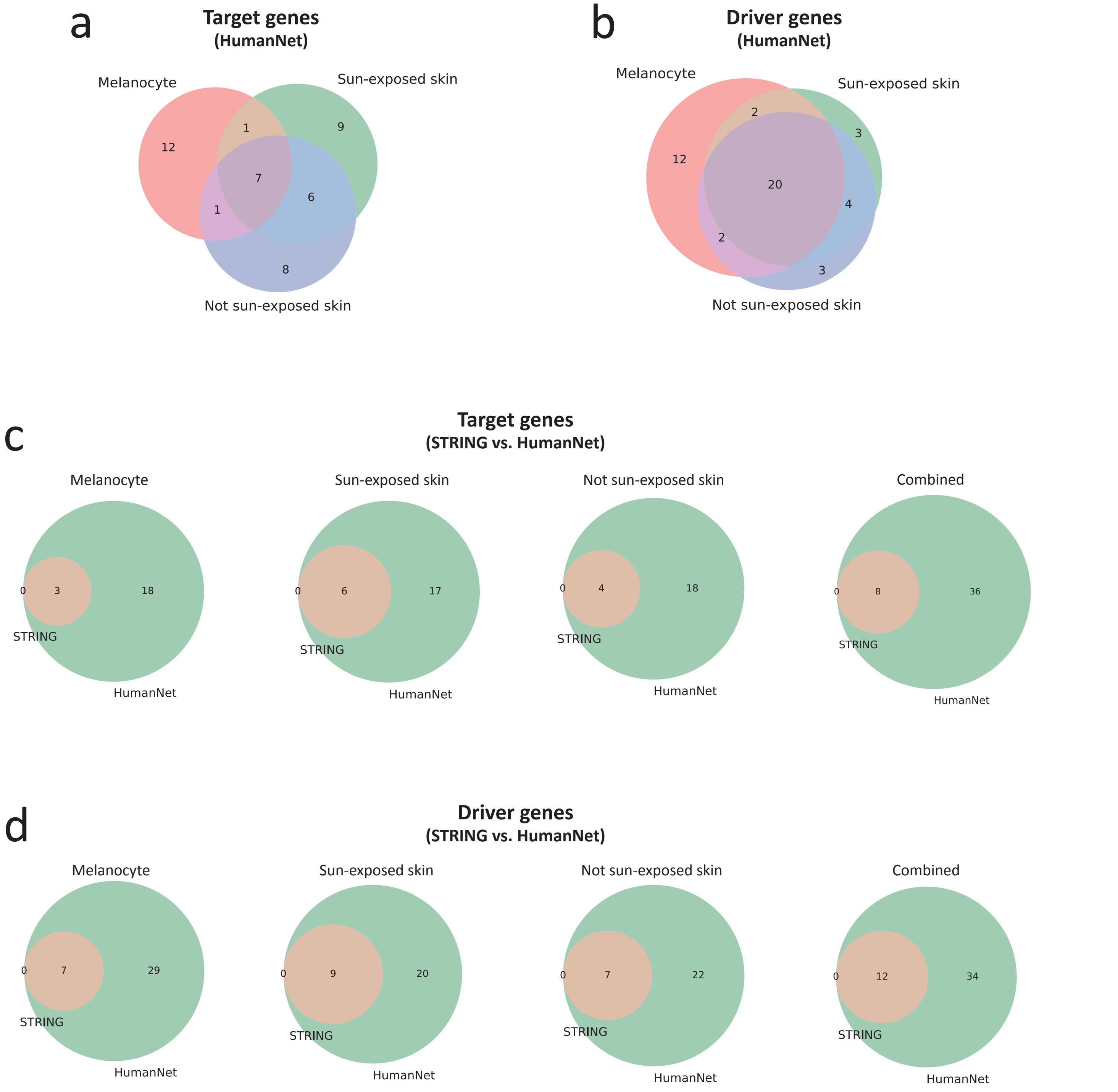

Supplementary Figure 4. Interaction between melanoma target genes and melanoma driver genes in HumanNet protein-protein interaction networks (PPINs). The overlaps of (a) melanoma target genes and (b) melanoma driver genes at level 1 within the HumanNet tissue-specific PPINs. (c) The overlaps between the melanoma target genes that interact with melanoma driver genes at level 1 in STRING and HumanNet PPIN for each tissue. (d) The overlaps between the melanoma driver genes at level 1 in STRING and HumanNet PPIN for each tissue.
