# Supplementary material for: Links between melanoma germline risk loci, driver genes and comorbidities: insight from a tissue‐specific multi‐omic analysis
Source: Mol Oncol. 2024 Feb 3;18(4):1031–48. doi: 10.1002/1878-0261.13599 (PMC10994230; doi:10.1002/1878-0261.13599)
Supplement: Supplementary file 5 — Fig. S5. Significant melanoma‐associated traits found using DisGeNET melanoma genes. [file MOL2-18-1031-s001.pdf]

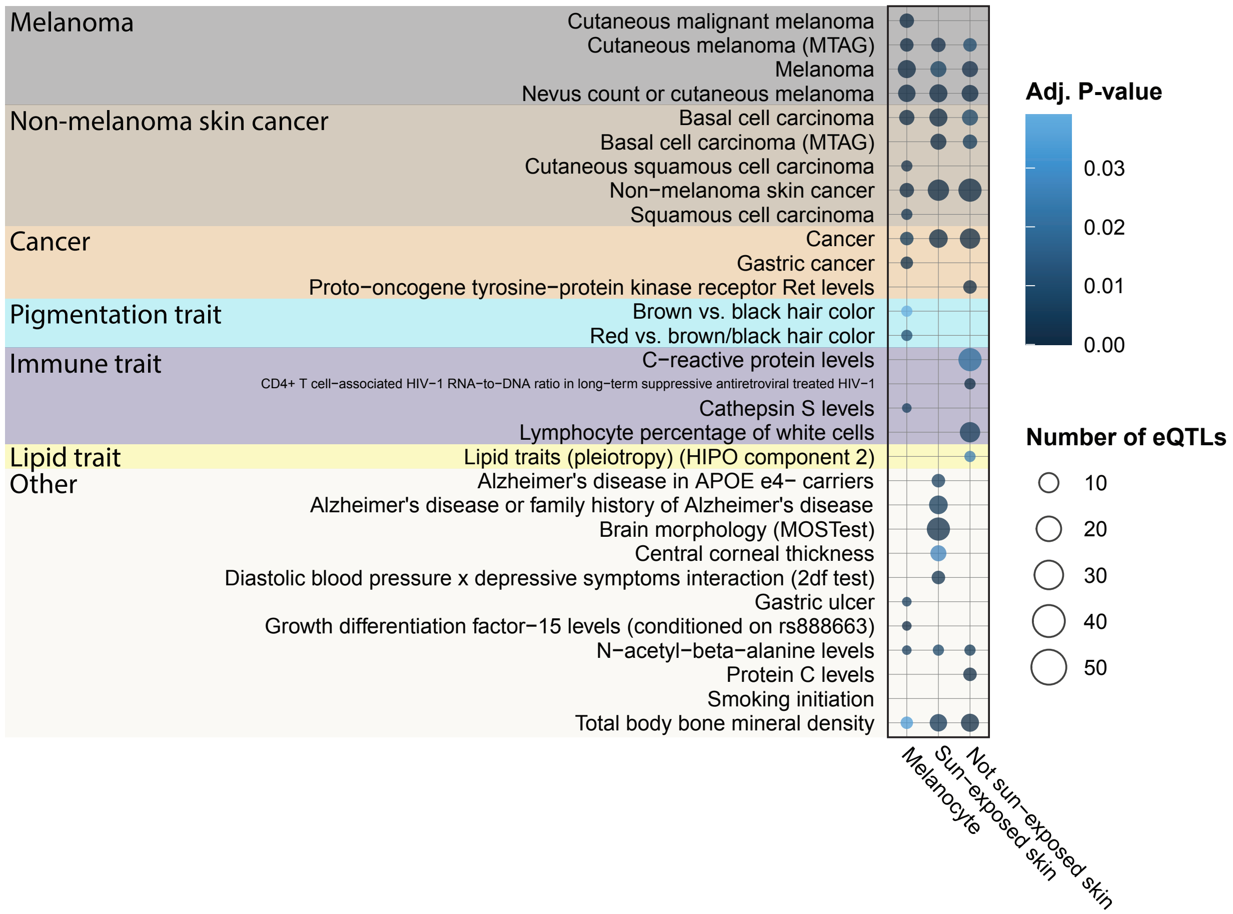

Supplementary Figure 5. Significant melanoma associated traits found using DisGeNET melanoma genes. Each gene regulatory network (GRN) was queried to identify the set of eQTLs in the GRN which targeted DisGeNET melanoma genes (n =248) instead of the respective tissue-specific melanoma target genes found in this study.
