# Supplementary material for: Links between melanoma germline risk loci, driver genes and comorbidities: insight from a tissue‐specific multi‐omic analysis
Source: Mol Oncol. 2024 Feb 3;18(4):1031–48. doi: 10.1002/1878-0261.13599 (PMC10994230; doi:10.1002/1878-0261.13599)
Supplement: Supplementary file 6 — Fig. S6. Hierarchical clustering shows the contribution of pleiotropic target genes to different associated phenotypes. [file MOL2-18-1031-s004.pdf]

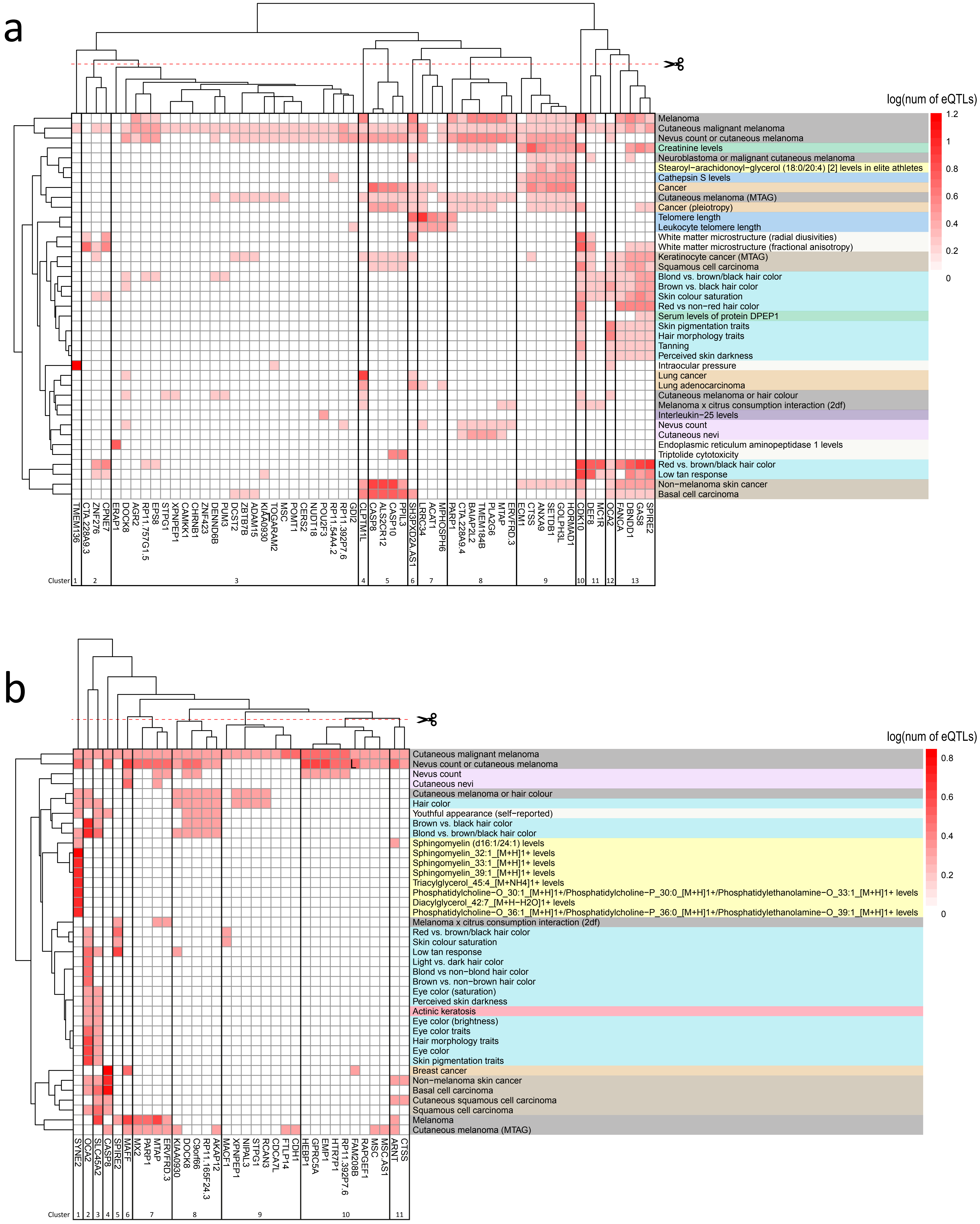

Supplementary Figure 6. Hierarchical clustering shows the contribution of pleiotropic target genes to different associated phenotypes. Hierarchical clustering was performed using the log number of GWAS SNPs of each trait that overlap with eQTL targeting the pleiotopic gene in question within linkage disequilibrium $r^2 > 0.8$ in (a) Not sun-exposed skin and (b) Melanocyte.
